# Reducing the Burn Marks on Injection-Molded Parts by External Gas-Assisted Injection Molding

**DOI:** 10.3390/polym13234087

**Published:** 2021-11-24

**Authors:** Jiquan Li, Wenyong Liu, Xinxin Xia, Hangchao Zhou, Liting Jing, Xiang Peng, Shaofei Jiang

**Affiliations:** 1 College of Mechanical Engineering, Zhejiang University of Technology, Hangzhou 310014, China; Lijq@zjut.edu.cn (J.L.); liuwenyonglwy@163.com (W.L.); sherlockingxia@163.com (X.X.); jinglt0805@zjut.edu.cn (L.J.); pengxiang@zjut.edu.cn (X.P.); 2 National International Joint Research Center of Special Purpose Equipment and Advanced Processing Technology, Zhejiang University of Technology, Hangzhou 310014, China; 3 Mechanical Light Industry Inspection Department, Zhejiang Fangyuan Test Group Co., Ltd., Hangzhou 310018, China; zhouhangchao@126.com

**Keywords:** burn mark, EGAIM, image processing, quantitative method, gas parameters

## Abstract

A burn mark is a sort of serious surface defect on injection-molded parts. In some cases, it can be difficult to reduce the burn marks by traditional methods. In this study, external gas-assisted injection molding (EGAIM) was introduced to reduce the burn marks, as EGAIM has been reported to reduce the holding pressure. The parts with different severities of burn marks were produced by EGAIM and conventional injection molding (CIM) with the same molding parameters but different gas parameters. The burn marks were quantified by an image processing method and the quantitative method was introduced to discuss the influence of the gas parameters on burn marks. The results show that the burn marks can be eliminated by EGAIM without changing the structure of the part or the mold, and the severity of the burn marks changed from 4.98% with CIM to 0% with EGAIM. Additionally, the gas delay time is the most important gas parameter affecting the burn marks.

## 1. Introduction

Injection molding is one of the most important mass production methods of plastic parts [1,2,3]. However, some defects commonly appear in injection-molded parts, such as flashes [4], warpage [5], weld lines [6], burn marks [7], sink marks [8], etc. Of the aforementioned defects, a burn mark is a kind of surface defect that shows a dark or black character in certain places on the part surface. This defect affects the appearance and mechanical properties of the molded parts greatly.

The influence of mold structure and molding parameters on the burn marks has been studied [9,10,11]. Yao et al. [9] proposed an automated design methodology to minimize the burn marks by optimizing the mold design, and the burn marks were eliminated with the optimized gate location. Mesgaran et al. [10] modified the mold structure to eliminate burn marks. The burn marks decreased with the modified mold. However, it is not always possible to modify the gate location or mold structure of injection molds. Fukushima et al. [11] investigated the influence of molding parameters on the burn marks and found that the burn marks decreased with a decrease in injection speed within a certain range. It provides guidance for reducing the burn marks, but it does not always work in the molding of all parts. Therefore, it is important to find a possible method of reducing the burn marks.

The burn marks are caused by high local temperatures of a polymer, usually due to the high pressure of trapped air in the mold cavity [11,12]. External gas-assisted injection molding (EGAIM) has been reported to reduce the holding pressure in the mold cavity [13,14,15], which will perhaps reduce the pressure at the air-trapped positions and hence reduce the burn marks.

EGAIM is more complicated than conventional injection molding (CIM) because of the introduction of gas [16]. The gas is injected between the mold wall and the molten polymer at a specific point in time (named the gas delay time) after filling with molten polymer, the gas will be maintained at a certain pressure (named the gas pressure) until a certain time (named the gas packing time) [15,17]. EGAIM compensates for the shrinkage of the polymer during the holding process better than that in conventional injection molding. EGAIM has been reported to mold thick-walled parts and uneven-thickness parts as it can compensate for the shrinkage and thus reduce the shrinkage [13], sink marks [16], ghost marks [17] etc. Additionally, the parts with higher surface quality can be molded and more materials can be saved with EGAIM.

In this study, EGAIM was introduced as a possible method to reduce the burn marks and the influence of EGAIM on burn marks were investigated in detail. The parts with different severities of burn marks were molded by the EGAIM and CIM processes. Images were taken of the molded parts and they were then divided into the burnt area and non-burnt area by image processing [18]. Thus, the burn marks were quantified by calculating the proportion of burnt area. The quantified burn marks of parts molded by EGAIM were compared with those by molded by CIM to see whether EGAIM is able to reduce the burn marks. Parts were also molded by the EGAIM process under different conditions with full factorial experiments, and quantified burn marks were also used to analyze the influence of gas parameters on the burn marks.

## 2. Materials and Methods

### 2.1. Experiment

**Figure 1:**
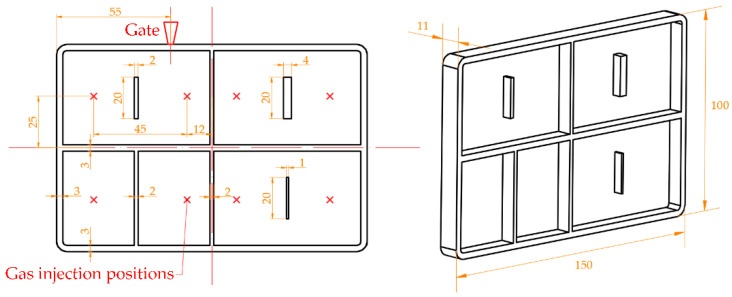
Dimensions of the parts and locations of the gate and gas injection positions (unit: mm).

In this study, the parts were molded by EGAIM and CIM with the same molding parameters except the special gas parameters in EGAIM. The burn marks were investigated on the molded parts to discuss whether EGAIM is applicable for reducing burn marks. The dimensions of the parts and locations of the gate and gas injection positions are shown in Figure 1. Additionally, the molding parameters used in EGAIM and CIM are shown in Table 1.

The special gas parameters in EGAIM are the aforementioned gas pressure, gas packing time and gas delay time. The full factorial experiment method is suitable to design experiments to study the effects of factors comprehensively, and it was introduced to investigate the influence of each gas parameter on burn marks. The levels of gas parameters used in the molding experiments are shown in Table 2.

A semi-crystalline iPP (Globalene 6331, Taiwan Polypropylene Co., Ltd., Taipei, China) was chosen as the part material in this study. An injection molding machine (MA3800/2250, Haitian International Holdings Ltd., Ningbo, China) was used to mold CIM and EGAIM parts. Nitrogen with a certain gas pressure was injected into the cavity controlled by a pressure controller (C8-01, Beijing Chn-Top Machinery Group Co., Ltd., Beijing, China).

### 2.2. Evaluation of Burn Marks

The parts with burn marks were photographed and the images were processed by an image processing method [18] designed to evaluate the burn marks of molded parts. The proportion of burnt area over the area of the whole part area was taken as the quantified index of burn marks in this paper.

The molded parts were put on clean backgrounds and the images were photographed on the surface with burn marks by a camera under stable light. The backgrounds were removed and the part images with burn marks were binarized according to [19,20,21] for calculating the proportion of burnt area.

In the binarization, a threshold was set to divide pixels in the image into black and white pixels, where black pixels exist only in the burnt area and white pixels exist only in the non-burnt area. The threshold was determined by the OTSU method [21]. The black and white pixels were converted by Python program, where the black pixels are the pixels with a gray level lower than the threshold and white pixels are the pixels with the gray level higher than the threshold. The number of black and total pixels in the binarized images are counted by the Python program, and the proportion of the burnt area *ε_b_* can be calculated by Equation (1):

(1) $$\varepsilon_{b}=\frac{n_{1}}{n}$$

where *n*_1_ is the number of black pixels in the binarized image, and *n* is the number of pixels in the binarized image.

### 2.3. Regression Analysis

Regression analysis is a useful method to investigate the relationships between variables [22,23,24] and it was introduced to quantify the influence of gas parameters on burn marks in this study. A model was established by stepwise regression analysis in Minitab to describe the relationship between the gas parameters and burn marks. The significance level was set to 0.05 and the goodness of fit was evaluated by the value of coefficient of determination, R^2^ and adjusted R^2^, R^2^_adjust_.

## 3. Results and Discussion

### 3.1. Quantification of Burn Marks

**Figure 2:**
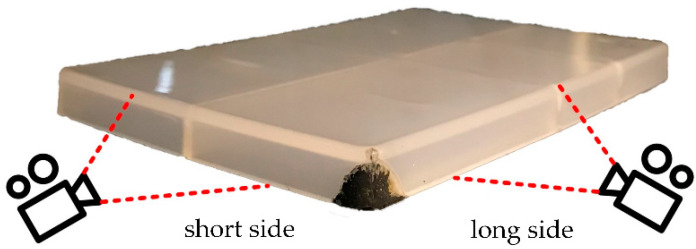
One of molded parts.

In this study, the burn marks appear at the final filling position, usually on the sides of the corner farthest from the injection gate. Additionally, the side with the length of 150 mm was marked as the long side and the one with the length of 100 mm was marked as the short side, as shown in Figure 2. The images were photographed on the long side and the short side, and image processing was performed on the images.

**Figure 3:**
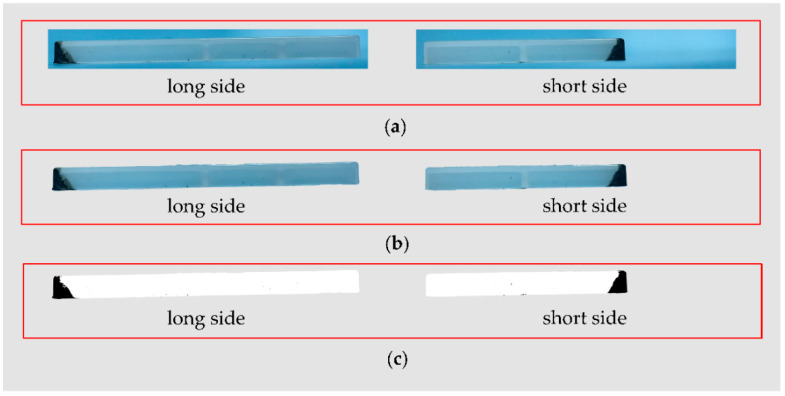
Images of main steps during the image processing of a part molded by CIM: (**a**) photographed images; (**b**) part images without background; (**c**) binary images.

The burn marks are very obvious on the parts molded by CIM. The image processing was performed on the CIM parts, and the images of the main steps during image processing are shown in Figure 3. The total pixels and black pixels in Figure 3c were counted by the Python program, and the proportions of burnt area were calculated on the long side and short side of the part, marked as *ε_l_* and *ε_s_*, respectively. The proportion of burnt area in part *ε* was defined as the proportion of burnt area on the sides with burn marks, namely the proportion of black pixels out of total pixels in the binary images. The *ε* values were calculated by Equation (2) in this study:

(2) $$\varepsilon =\frac{3\times \varepsilon_{l}+2\times \varepsilon_{s}}{5}$$

*ε* = 4.98% for the parts molded by CIM with the molding parameters shown in Table 1.

**Figure 4:**
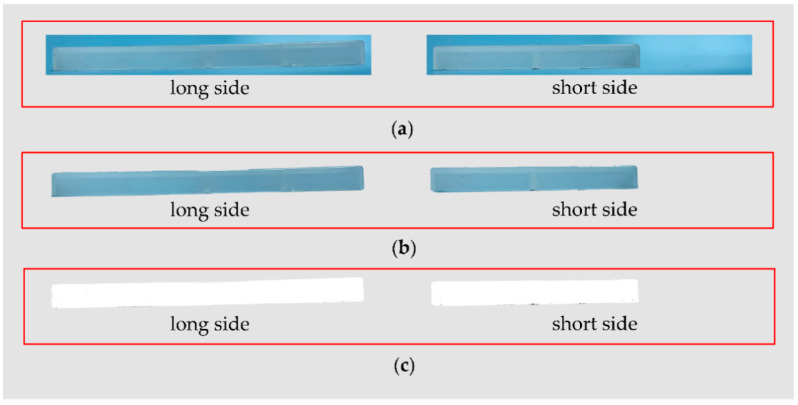
Images of the main steps during the image processing of a part molded by EGAIM: (**a**) photographed images; (**b**) part images without background; (**c**) binary images.

Burn marks are invisible in many parts molded by EGAIM, and one of the parts was selected to quantify the burn marks by image processing. This part was molded with a gas pressure of 7 MPa, gas packing time of 30 s, and gas delay time of 0 s. Images of the main processing steps of the molded part are shown in Figure 4. Additionally, the proportion of burnt area is 0 in this part with invisible burn marks.

In Figure 3, the black area in the binary images, shown in Figure 3c, is basically the same as the burnt area on the parts shown in Figure 3a. Additionally, in Figure 4, the burnt area on the part is invisible and the black area in the binary images is also invisible. Therefore, the binarization is effective when comparing the part images with the binary images.

The proportions of burnt area in the two aforementioned parts are very different from each other in terms of the severity of the burn marks. Thus, the proportion of burnt area is an effective quantifiable index to evaluate the burn marks.

### 3.2. Reduction in Burn Marks by EGAIM

To discuss whether EGAIM is effective at reducing burn marks, the severities of burn marks in parts molded by EGAIM were compared with those molded by CIM. The EGAIM and CIM parts were molded with the same molding parameters as shown in Table 1, except the gas parameters used in EGAIM were different. The gas parameters were set by full factorial experiments with levels as shown in Table 2. The severities of burn marks were quantified by the aforementioned image processing method. The severities of burn marks of parts molded by EGAIM, with detailed gas parameters are listed in Table 3.

**Figure 5:**
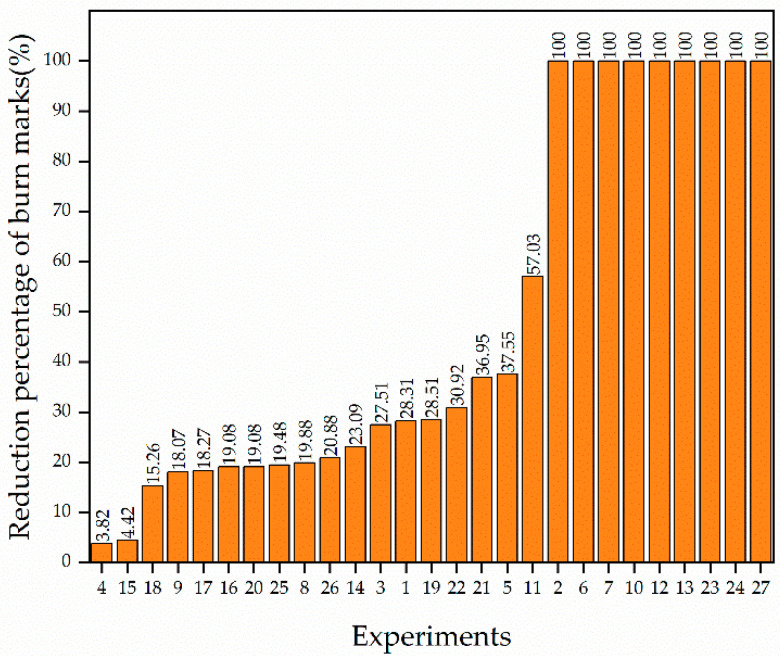
Reduction percentage of burn marks in parts molded by EGAIM.

The proportions of burnt area in the parts molded by EGAIM are all lower than the 4.98% burn rate observed in CIM, as shown in Table 3. Therefore, EGAIM is an effective method to reduce burn marks, as we predicted in the introduction. The severity of burn marks varied very much with different gas parameters. To discuss the reduction in burn marks by EGAIM more comprehensively, the reduction percentages of burn marks are ranked from lowest to highest according to the data listed in Table 3, as shown in Figure 5.

In one third of the experiments, the burn marks were completely invisible on the molded part. The burn marks were eliminated by EGAIM with the given gas parameters in those experiments. More than half of the experiments show reduction percentages from 15 to 40%, and the lowest reduction percentage is about 4%, appearing in only two experiments. The burn marks were reduced to a certain degree by EGAIM.

### 3.3. Influence of Gas Parameters on Burn Marks

**Figure 6:**
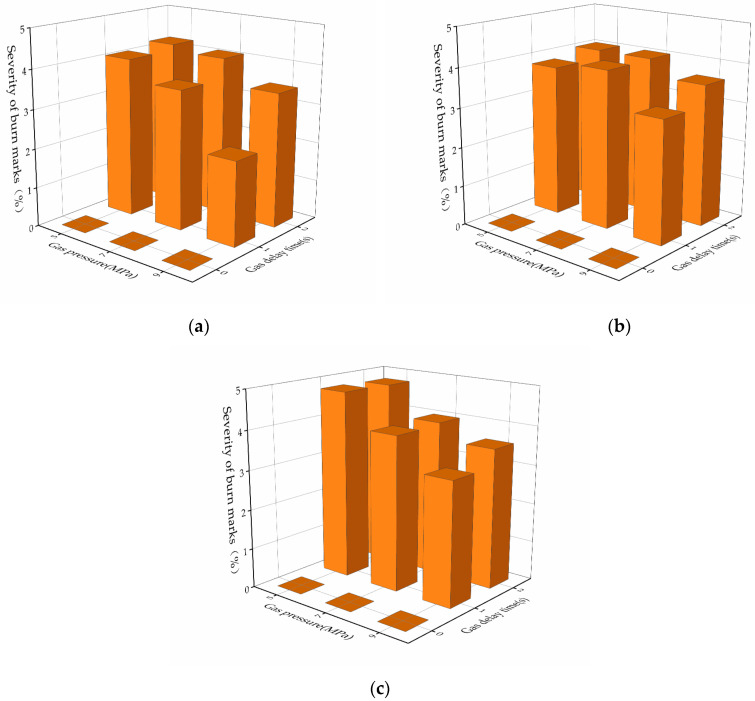
Influence of gas pressure and gas delay time on burn marks: (**a**) gas packing time of 10 s; (**b**) gas packing time of 20 s; (**c**) gas packing time of 30 s.

**Figure 7:**
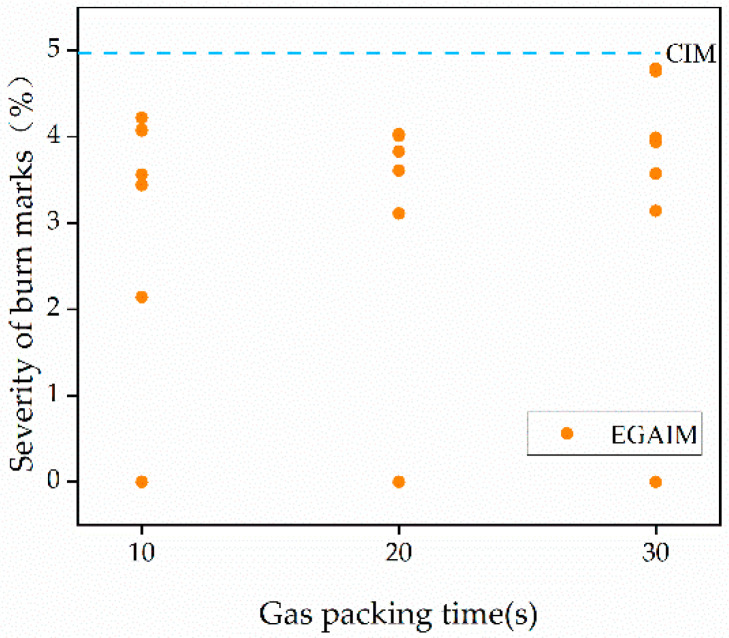
Influence of gas packing time on burn marks.

The reduction in burn marks varied very much with different gas parameters, as discussed in Section 3.2. Thus, it is necessary to investigate the influence of gas parameters on the reduction in burn marks. Three-dimensional bar plots were created to show the influence of gas pressure and gas delay time on burn marks with different gas packing times, and a scatter plot was used to show the influence of gas packing time on burn marks, as shown in Figure 6 and Figure 7.

Figure 6 clearly shows that the burn marks are reduced to 0 when the gas delay time is 0 s. A gas delay time of 0 s means that the gas is injected between the cavity surface and molded part at the end of the filling immediately. The sooner the gas is injected, the sooner it will reduce the holding pressure, and thus this significantly reduces the severity of the burn marks within this study. In addition, the severities of burn marks decrease gradually with an increase in gas pressure from 5 MPa to 9 MPa. Compared with lower gas pressure, higher gas pressure reduces the holding pressure more, reducing burn marks more significantly.

In Figure 7, the dots correspond to the severities of burn marks of parts molded by EGAIM with different gas parameters, and the dashed line corresponds to the severity of the burn marks of parts molded by CIM. The influence of gas packing time on burn marks cannot be observed clearly in Figure 7. It will be discussed specifically in the following section.

### 3.4. Regression Analysis for the Gas Parameters and Burn Marks

**Figure 8:**
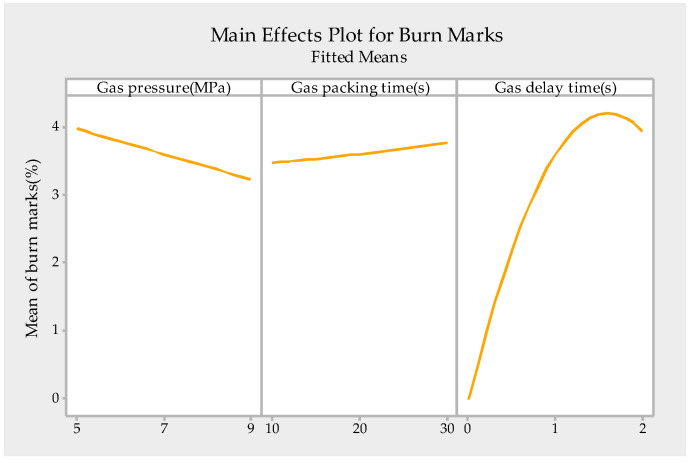
Main effects plot for burn marks.

A stepwise regression analysis was introduced to establish the model of the relationship between gas parameters and burn marks. The regression model is shown in Table 4 and the main effect plot for burn marks is shown in Figure 8.

According to the F-distribution table, F_0.05_(6,20) = 2.6. Additionally, Table 4 shows that F = 122.16 > F_0.05_(6,20), indicating the model is significant at a significance level of 0.05. Additionally, the values of R^2^ and R^2^_adjust_ are 97.34% and 96.55%, respectively, which are close to 1. The goodness of fit for this model is much higher than the goodness of fit reported in the literature with regression analyses [16,24,25,26]. Thus, the model accurately describes the relationship between the gas parameters and burn marks, and it is acceptable to discuss in more detail based on the result of regression analysis, as shown in Figure 8.

Figure 8 shows that the gas delay time is the most important parameter of the gas parameters influencing the burn marks, followed by the gas pressure, and the gas packing time is the least important gas parameter influencing the burn marks. The burn marks can be greatly reduced by adjusting the gas delay time, whereas they can be slightly reduced by adjusting the gas pressure and gas packing time.

## 4. Conclusions

In this study, EGAIM was introduced to reduce burn marks and the influence of gas parameters on burn marks was studied. The parts were molded by CIM and EGAIM, and different severities of burn marks appeared on the molded parts. The severities of burn marks were quantified by the image processing method. The quantified burn marks of parts molded by EGAIM were compared with those molded by CIM and were used to discuss the influence of the gas parameters on burn marks. The following conclusions can be drawn:

(1) EGAIM is an applicable process to reduce burn marks in injection molding without changing the structure of the part or the mold. The proportions of burnt area in parts molded by EGAIM were all lower than the 4.98% that was found for those molded by CIM, and the burn marks could be eliminated by EGAIM with reasonable gas parameters.
(2) The burn marks were quantified into specific values by the quantitative method proposed in this paper, and this provides a possible method for evaluating other visible defects in injection-molded parts.
(3) Different gas parameters play different roles in reducing burn marks. The most important gas parameter is gas delay time. The burn marks were eliminated when the gas delay time was 0 s and varied slightly with changes in gas pressure and gas packing time.

## Figures and Tables

**Table 1:** The molding parameters used in EGAIM and CIM.

Melt Temperature (°C)	Mold Temperature (°C)	Injection Pressure (MPa)	Injection Speed (mm/s)	Holding Pressure (MPa)	Holding Time (s)	Cooling Time (s)
220	40	60	55	50	5	35

**Table 2:** Gas parameters and levels.

	Level 1	Level 2	Level 3
Gas pressure (MPa)	5	7	9
Gas packing time (s)	10	20	30
Gas delay time (s)	0	1	2

**Table 3:** The detailed gas parameters and severities of burn marks of parts molded by EGAIM.

No.	Gas Pressure (MPa)	Gas Packing Time (s)	Gas Delay Time (s)	Severity of Burn Marks (%)
1	9	30	2	3.57
2	5	30	0	0
3	9	20	2	3.61
4	5	30	1	4.79
5	9	20	1	3.11
6	7	10	0	0
7	7	20	0	0
8	7	30	2	3.99
9	5	10	1	4.08
10	9	30	0	0
11	9	10	1	2.14
12	9	10	0	0
13	5	20	0	0
14	5	20	1	3.83
15	5	30	2	4.76
16	7	20	2	4.03
17	7	10	2	4.07
18	5	10	2	4.22
19	7	10	1	3.56
20	5	20	2	4.03
21	9	30	1	3.14
22	9	10	2	3.44
23	9	20	0	0
24	5	10	0	0
25	7	20	1	4.01
26	7	30	1	3.94
27	7	30	0	0

**Table 4:** Regression model between gas parameters and burn marks.

The Model Established in Minitab	*p*-Value	F-Value	R^2^	R^2^_adjust_
Severity of burn marks = 0.393 − 0.0865 Gas pressure + 0.0047 Gas packing time + 5.957 Gas delay time + 0.00026 Gas packing time × Gas packing time − 1.638 Gas delay time × Gas delay time − 0.0996 Gas pressure × Gas delay time	1.1 × 10^−14^	122.16	97.34%	96.55%

## Data Availability

Data sharing is not applicable to this article.
